# Where in 1900?

**Published:** 1899-11

**Authors:** 


### EDITORIAL.

### WHERE IN 1900?

The race for the place of meeting in 1900 grows more interesting as the time approaches for the decision by the Executive Committee. Appeals are strong for some three of the cities, and the final decision will be awaited with much interest.

Detroit has strong claims for the place of meeting, in that she has waited for its coming for several years, and her commercial interests are ever ready to encourage convention gatherings of every kind. Her commercial interests appreciate full well the value of bringing strangers within her gates, that they may learn of her attractions, progress, and power in the business world. Her veterinarians are active and well known, and, though she steps out of the field as a centre of veterinary learning, she has given of her best to sustain these interests and awakened a higher appreciation of the veterinary profession than ever before.

Her wide commercial pursuits have fostered and encouraged the growth of closely allied interests in the field of medicine and given much in highly valued products that have contributed a deal to the progress of scientific medicine. That her veterinarians would welcome our coming needs no affirmation, and had our influence been felt there in the past, the State and the city, of which she is justly proud, might have written a different history in the annals of our progress in college work and legislation.

Of Columbus much may be said in favor of her geographical location in the Central West, of her railroad facilities, of the large numbers she has contributed to the veterinary profession, many of whom have entered State and National veterinary work and won honor and fame for her devotees of their calling. Ohio's progress in State veterinary sanitary work has been changeful and uncertain; her efforts in college direction have been of slow growth, and the many changes in teaching staffs marred the true progress of college building, and she lingers yet in the field of college work with records of one misguided undertaking that proved a drawback to our progress. These directions of veterinary efforts would no doubt be given a strong impetus by our meeting there, and new life, fresh energy, and increased public interest developed to sustain them along a higher and better plane, more in touch with the aims and purposes of our national organization.

Not unmindful of the claims of these rival cities, Minnesota points the way and extends the hand of welcome to her beautiful twin cities of the North. She strongly urges our coming, knowing full well the value of our temporary sojourn in her midst. The record of her membership in our National organization is a proud one, and the loyalty, interest, and support received from her membership is a shining mark upon her escutcheon. She, too, has made history in having crossed the portals of college work, but ever desirous of leading in aught that she has done, her zeal and interest, her confidence in the distant future were too much in advance of the hour and business depression that visited her chief commercial centres, forced to the wall these well-projected colleges of advanced curricula, and she stepped from the field of work with a clean record of true efforts to meet the needs of the hour. Better would it have been had such been the outcome in other fields that have lacked the courage of Minnesota's pioneers, and remained in the field to close or maintain their efforts in college direction to their own discredit and dishonor, not to measure what they have done in adding to the difficulties and labors of those who have sustained the demands of our national body in the educational field.

Her strength in numbers and attendance upon meetings of our national body has grown with great rapidity the past five years.

The State she so well represents is moving with wonderfully progressive strides in the field of veterinary sanitary work. The education of those engaged in the live-stock industries, the development of public sentiment to sustain the true progress of preventive medicine, makes this a record-making epoch of advancement that reflects much honor upon her representative veterinarians.

In safeguards for her people from impostors, quacks, and ignorant pretenders, who live as parasites of the profession ; in the establishment and maintenance of wise laws and regulations for the practice of veterinary medicine, she stands in the forefront.

She seeks our coming that her hands and efforts in these and other directions of great public import may be strengthened and sustained. She realizes that our presence will be a factor in awakening wider public interest in these measures, and, through her many avenues of quick communication, throughout the great Northwest, set in motion centres of quickened activity that will make possible and easier the task of solving these great questions of so much import to our calling.

She deserves well at the hands of the profession, and their appeals should not be unheard by the members of the Executive Committee in her behalf. Her appeal comes with peculiar force at an opportune moment, and a favored decision would ensure a successful meeting, grander in results than we can conjecture to-day, and a fitting response to the claims of a section of our broad land that needs our every assistance and encouragement.

---

Ex-Chairman Williams, of the Publication Committee, joins with the Journal in a regretful protest that too much of our convention time is given to the social features and round of festivities afforded us by the local veterinarians. He refers to the almost total monopoly of the time of the local Committee of Arrangements, so that our conventions come and go without any of these bodies having any adequate idea of the work done or its transactions. He urges as one remedy one entire afternoon and one evening for the social end, evening sessions of the Association, and sectional work as another feature. The editors of this Journal wholly approve of the evening sessions, the curtailing of the round of pleasures, but we are not yet ready for sectional work. There are yet too many aspects of our work with which every veterinarian must be more or less acquainted to be successful in his vocation. There is but one field yet sufficiently developed for consideration from this point, and that is State medicine, and we would urge one day, or at least one session, for the consideration of this work. We do not feel that the importance of this field of our work receives as yet the attention it well merits, and if we are to fully secure for our calling its proper recognition, we must give this subject the fullest consideration.

The Journal again asks, What will the Executive Committee do?

